# The Effect of 6-Thioguanine on Alternative Splicing and Antisense-Mediated Exon Skipping Treatment for Duchenne Muscular Dystrophy

**DOI:** 10.1371/currents.md.597d700f92eaa70de261ea0d91821377

**Published:** 2012-12-12

**Authors:** Ingrid E. C. Verhaart, Annemieke Aartsma-Rus

**Affiliations:** Department of Human Genetics, Leiden University Medical Center, Leiden, The Netherlands; Department of Human Genetics, Leiden University Medical Center, Leiden, The Netherlands

## Abstract

The severe muscle wasting disorder Duchenne muscular dystrophy (DMD) is caused by genetic defects in the DMD gene, leading to a complete absence of dystrophin protein. Of the therapeutic approaches addressing the underlying genetic defect, exon skipping through antisense oligonucleotides (AONs) is the closest to clinical application. Several strategies to improve the efficiency of this approach are currently being investigated, such as the use of small chemical compounds that improve AONmediated exon skipping levels. Recently, enhanced exon skipping in combination with a guanine analogue, 6-thioguanine (6TG) was reported for phosphorodiamidate morpholino oligomers (PMO). Here the effect of 6TG on the exon skipping efficacy of 2’-O-methyl phosphorothioate RNA (2OMePS) and PMO AONs in vitro and in vivo was further evaluated, as well as the effect of 6TG by itself. Results confirm an increase of exon skipping levels in vitro, however, in contrast to the previous report, no effect was observed in vivo. Importantly, 6TG treatment in vitro resulted in numerous additional DMD exon skipping events. This, in combination with the known cytotoxic effects of 6TG after incorporation in DNA, warrants reconsidering of the use of 6TG as enhancer of AON efficiency in DMD, were chronic treatment will be required.

## Introduction

Duchenne muscular dystrophy (DMD) and Becker muscular dystrophy (BMD) are both muscle wasting disorders caused by mutations in the *DMD *gene. However, DMD is characterized by a much more severe and progressive phenotype than BMD. In DMD, children generally become wheelchair-dependent around the age of ten, need assisted ventilation before their twenties, develop cardiac problems shortly thereafter and die before their thirties. By contrast, the BMD phenotype is much more variable with an average age of onset around 12, wheelchair-dependency in their twenties and death in their forties for the most severely affected patients^1^. However, less severely affected patients can remain asymptomatic into their fifties and have nearly normal life expectancies^2^. Furthermore, only around 50% of BMD patients develops dilated cardiomyopathy^1^. The differences between both phenotypes are due to the different nature of the underlying genetic defects. The *DMD *gene, located on Xp21, encodes dystrophin, an important protein for muscle fibres. Dystrophin forms a bridge between the actin cytoskeleton and the extracellular matrix, thereby giving mechanical stability to the fibres during contraction^3^. In DMD the reading frame is disrupted, leading to a premature stop of translation and thereby formation of a truncated, non-functional protein. Consequently, fibres are easily damaged and die, which leads to a gradual loss of muscle tissue and replacement by fibrotic and adipose tissue. By contrast, in BMD mutations do not affect the reading frame, allowing protein translation to continue, resulting in internally deleted dystrophin proteins, which are partly functional. Therefore in BMD patients the disease progression is slower^4^.

This difference underlies the rationale of the exon skipping therapy. Hereto antisense oligonucleotides (AONs) are used to hide a specific exon from the splicing machinery, inducing the skipping of this exon in order to restore the reading frame and allow the production of a shortened, Becker-like dystrophin protein. After demonstration of proof-of-principle and obtaining encouraging results *in vitro *in cultured patient-derived cells and *in vivo *in animal models, clinical trials are currently ongoing^5^. For these trials two different backbone chemistries are used: 2’-*O*-methyl phosphorothioate RNA (2OMePS) and phosphorodiamidate morpholino oligomers (PMO).

There are several ways to further optimize the efficiency of AON-mediated exon skipping. First, AON efficiency can be improved following previously identified guidelines that take into account target RNA accessibility, thermodynamic properties and the presence of known splicing motifs^6^. Furthermore the efficiency depends on the amount that reaches its target organ (muscle and heart) and organelle (cell nucleus), but also on the turnover of the compound, skipped transcripts and internally deleted dystrophin proteins, and on the muscle cells. A second approach has been to improve AON efficacy by conjugating PMOs to cell-penetrating peptides (PPMOs) or dendrimeric octaguanidine polymers (vivo-morpholinos)^7^ ^8^. These indeed lead to enhanced exon skipping and protein restoration, but also raise safety concerns^9^. Determination of a relatively low LDR50R of PPMOs indicates a high level of acute toxicity^10^. A third strategy is to optimize administration or dosing regimens (Verhaart et al., manuscript submitted). Another possibility is to enhance exon skipping levels using small chemical compounds. Chemical compounds that influence splicing have been reported, i.e. the cytokine kinetin to specifically correct the splice defect in familial dysautonomia and several compounds have been identified to enhance exon 7 inclusion in spinal muscular atrophy^11^ ^12^. For DMD, TG003 has been reported to induce exon 31 skipping and dystrophin restoration in patient cells harbouring a point mutation in this exon^13^. However this compound is specific for this mutation, so its application is rather limited. In order to identify more general exon skipping enhancing compounds, large drug screening systems have been used. Ideally these compounds should enhance AON-induced exon skipping rather than inducing skipping by themselves, as the latter involves the risk of aspecific exon skipping events. For DMD, Hu et al. indentified a guanine analogue, 6-thioguanine (6TG) that enhances PMO-induced exon 23 skipping *in vitro *in cultured *mdx *cells, which carry a nonsense mutation in exon 23, thereby causing the absence of dystrophin protein^14^. Increased exon skipping levels were also observed after local intramuscular injection of AONs and 6TG in *mdx *mice^15^.

6TG is widely known as an antileukemic agent due to its cytotoxic effects after incorporation in the DNA during replication^16^. The mechanism by which 6TG enhances PMO-induced exon skipping on pre-mRNA level is not known. Hu et al. suggested that alternative splicing could be enhanced after alteration of the structure of the *DMD *gene by incorporation of 6TG. Alternatively, 6TG, a small nucleobase, might interact with the bases of the PMO, thereby improving delivery efficiency to the nucleus and binding to the targeted sequence^15^.

To investigate whether the effect of 6TG on exon skipping also applies to other exons and AON chemistries, here the effects of 6TG treatment alone or in combination with AONs targeting several human exons *in vitro *and mouse exon 23 *in vitro *and *in vivo *were examined. *In vitro *6TG indeed enhanced exon skipping levels for all targeted exons, but only at low AON concentrations or with suboptimal AON sequences. However, we were not able to detect the previously reported, enhanced exon skipping levels for 2OMePS or PMO AONs. Furthermore, *in vitro *6TG treatment resulted in numerous additional, unintended *DMD *exon skipping events.

## Materials and methods

**Cell culture**

An immortalized control human myoblast cell line 7304.1^17^, a mouse myoblast cell line C2C12 and primary DMD patient-derived myoblasts cultures with a deletion in exon 51-55 (DL589.2)^18^ were used in this study. Cells were cultured on a collagen layer (1:30; Pure Col; Nutacon BV; The Netherlands). DL589.2 cells were cultured in Nutrient Mix F-10 (HAM) supplemented with GlutaMAX, 20% fetal bovine serum (FBS) and 1% Penicillin-Streptomycin (P/S) (Gibco-BRL; the Netherlands). For 7304.1 cells this medium was mixed 1:1 with Skeletal Muscle Cell Basal Medium supplemented with 10% FBS, 1.4% GlutaMAX, 1% P/S, 5 μg hEGF, 0.5 μg hFGF, 25 mg Fetuin, 5 mg Insulin and 200 μg Dexamethasone (PromoCell GMbH; Germany). C2C12 cells were cultured in Dulbecco's Modified Eagle Medium (DMEM, without phenol red) supplemented with 10% FBS, 1% P/S, 2% GlutaMAX and 1% D-glucose (Gibco-BRL). Human cells were grown at 37 °C, 5% COR2R and mouse cells at 37 °C, 10% COR2R. To induce differentiation into myotubes, medium was changed into DMEM supplemented with 2% FBS, 1% P/S, 2% GlutaMAX and 1% D-glucose (Gibco-BRL) for C2C12 and patient-derived cells and into DMEM supplemented with 3% horse serum (Gibco-BRL), 1% P/S and 2% GlutaMAX for 7304.1 cells when cells were 80-90% confluent. Cells were differentiated in 3 mL differentiation medium per well of a 6-wells plate for 5 to 10 days depending on the cell type.

**6TG treatment and AON transfection**

6-Thioguanine (6TG; Cas number 154-42-7; Sigma-Aldrich; the Netherlands) was added 48 hours before AON transfection and maintained during and after transfection at indicated concentrations (30-90 μM). For 2OMePS AONs (Eurogentec; Belgium and Prosensa Therapeutics; the Netherlands) cells were transfected in 1 mL differentiation medium for 4 hours with indicated concentrations using polyethylenimine (PEI, Exgen 500; MBI Fermentas; Germany), according to the manufacturer’s instructions, using 2 μL of PEI per μg of AON for human cells and 3.5 μL of PEI per μg of AON for mouse cells. Cells were harvested >24 hours after transfection. For PMO AON (Gene Tools; OR, USA) experiments, PMOE23(+07-18) was added 72 hours before RNA analysis with or without 6TG at indicated concentrations according to manufacturer’s instructions. An overview of used AONs, their backbone chemistries and sequences is given in table 1.

**Table 1: Overview of AONs and their sequence used in this study:** P2OMePS = 2’-O-methyl phosphorothioate RNA; PMO = phosphorodiamidate morpholino oligomers

AON name	Target species	Chemistry	Sequence (5'-3')
h45AON5	human	2OMePS	gcccaaugccauccugg
h50AON1	human	2OMePS	cucagagcucagaucuu
h50AON2	human	2OMePS	ggcugcuuugcccuc
h53AON1	human	2OMePS	cuguugccuccgguucug
h55AON1	human	2OMePS	cuguugcaguaaucuaugag
M23D	mouse	2OMePS	uccauucggcuccaaaccgg
PMOE23	mouse	PMO	ggccaaaccttcggcttacctgaaat

***In vivo* 23AON and 6TG treatment**

All experiments were approved by and performed following the guidelines of the Dier Experimenten Commissie (Animal Experimental Commission) of the Leiden University Medical Centert (permit number: 08224). Effort was put in minimizing the amount of distress caused to the animals as much as possible. Mice were housed in individually ventilated cages in the animal facility of the LUMC and received food and drink *ad libitum*. *Mdx *mice (C57Bl/10ScSn-DMDP *mdx *P/J) were obtained from our own breeding facility.

For the first experiment 5 weeks old *mdx *mice were anesthetized with isoflurane. Mice were intramuscularly injected in both gastrocnemius muscles on 2 consecutive days with 0, 1, 10 or 50 μg 6TG with or without 20 μg (≡2.9 nmol) M23D(+2–18) 2OMePS AONs specifically targeting exon 23^19^ (Prosensa Therapeutics) in 40 μL saline (n=2 per condition; 6TG dosing was kept constant within one mouse). Mice were sacrificed 10 days after the last injection by cervical dislocation and muscles were isolated.

For the second experiment 7 or 8 weeks old *mdx *mice were anesthetized with isoflurane and intramuscularly injected in both gastrocnemius muscles on 2 consecutive days with 20 μg (≡2.9 nmol) M23D(+2–18) 2OMePS AON, or 5 μg (≡0.60 nmol) M23D(+07-18) PMO AON with or without 25 μg 6TG or 25 μg 6TG only (n=4 per condition; 6TG dosing was kept constant within one mouse). Mice were sacrificed 10 days after the last injection by cervical dislocation and muscles were isolated.

**RNA extraction and analysis of exon skipping by RT-PCR**

Harvested cells were lysed with TriPure isolation reagent (Roche Diagnostics; Switzerland). Total RNA was extracted and 400 ng of RNA was used for RT-PCR analysis, using Transcriptor reverse transcriptase polymerase (Roche) in 20 μL at 55 °C for 30 min with an appropriate primer (primer sequences on request). cDNA was amplified by nested PCR. Three microliters of cDNA were amplified in a 25 μL reaction for 20 cycles of 94 °C (40 sec), 60 °C (40 sec) and 72 °C (80 sec), followed by 32 cycles of 94 °C (40 sec), 60 °C (40 sec) and 72 °C (60 sec), with 1.5 μL of PCR product in a 50 μL reaction.

Muscles were homogenized in TriPure isolation reagent, using zirconium beads (1.4 mm; OPS Diagnostics; NJ, USA) by grinding in a MagNA Lyser (Roche) according to manufacturer’s instructions. Total RNA was extracted and 1 μg of RNA was used for RT-PCR analysis, using Transcriptor reverse transcriptase polymerase in 20 μL at 42 °C for 45 min with random hexamer primers (20 ng/μL). Then, 1.5 μL of cDNA was amplified in a 50 μL reaction for 30 cycles of 94 °C (30 sec), 60 °C (30 sec) and 72 °C (30 sec), as previously described^20^. All PCR products were visualized on 1.5 or 2% agarose gels and exon skipping levels were quantified using a DNA 1000 LabChip on the Agilent 2100 bioanalyzer (Agilent Technologies; CA, USA) according to the manufacturer’s protocol.

**Sequence analysis**

RT-PCR products were isolated from agarose gels using the QIAquick Gel Extraction kit (Qiagen; the Netherlands) according to manufacturer’s instructions. Direct DNA sequencing was performed by the Leiden Genome Technology Center (Leiden, the Netherlands) using the BigDye Terminator Cycle Sequencing Ready Reaction kit (PE Applied Biosystems) and analyzed on a 3730*xl *DNA Analyzer (PE Applied Biosystems).

**Protein extraction and Western blot analysis**

Muscles were minced in treatment buffer containing 75 mM Tris-HCl pH 6.8-15% (w/v) sodium dodecyl sulphate (SDS) using zirconium beads (1.4 mm; OPS Diagnostics) by grinding in a MagNA Lyser. Protein concentrations were determined using a Pierce bicinchoninic acid (BCA) protein assay kit (Thermo Fisher Scientific; MA, USA) according to manufacturer’s instructions. Samples containing 30 μg of protein were made in treatment buffer with 20% (v/v) glycerol, 5% (w/v) β- Mercaptoethanol and 0.001% (w/v) Bromophenol blue and heated for 5 min at 95 °C. As reference, wild type (C57Bl/10ScSnJ) control samples containing 33%, 11%, 3.7%, 1.2% and 0.4% of protein were used. Samples were loaded on 1.0 mm thick native PAGE Tris-acetate (polyacrylamide) gels, with a linear resolving gel gradient of 3-8% (Bio-Rad; the Netherlands) and run on the Trans-Blot Turbo system for 1 h at 75 V (~0.07 A) and 2 hrs at 150 V (~0.12 A) in XT Tricine running buffer (Bio-Rad) in an ice container (Hulsker et al., manuscript in preparation). Ready to use Trans- Blot Turbo transfer packs in combination with the Trans-Blot Turbo transfer system (BioRad) at 2.5 A and ~25 V for 10 minutes were used to blot the proteins on a nitrocellulose membrane, which were blocked in 10 mM Tris-HCl (pH 8) and 0.15 M NaCl (TBS)-5% non-fat dried milk (Elk, Campina Melkunie; the Netherlands) and washed in TBS-0.05% (v/v) Tween20 (TBST). Membranes were incubated overnight with primary antibodies 1:125 NCL-Dys1 (1:125; Dy4; NovoCastra,;T UKT) and α-actinin (loading control; 1:5000; AB72592; Abcam; UK) in TBS. Membranes were washed in TBST, incubated 1 h with the fluorescent secondary antibodies IRDye 800CW goatαmouse IgG (1:5000; Li-Cor; NE, USA) and IRDye 680LT donkeyαrabbit IgG (1:10 000; Li-Cor) in TBS, washed in TBST and TBS and analysed with the Odyssey system and software (Li-Cor).

## Results

**6TG induces exon skipping by itself and enhances exon skipping by 2OMePS AONs in human muscle cells *in vitro***

The effect of 6TG on exon skipping by 2OMePS AONs was first tested *in vitro *in cultured human muscle cells derived from a healthy control. Without 6TG exon 50 skipping was observed in decreasing levels till 50 nM of h50AON1. When 6TG was added, exon skipping was also observed for lower levels (25 and 5 nM) of h50AON1 and without AON. 6TG enhanced exon skipping levels for 50 nM of AON, but not for higher AON concentrations (100 and 200 nM) (fig. 1a). For a less efficient AON (h50AON2) again low exon skipping levels in combination with 6TG were observed for 0-25 nM of AON. However, for higher AON concentrations (50-200 nM) a clear enhancement was observed in combination with 6TG compared to h50AON2 only (fig. 1b). The same effect was seen for the AONs h45AON5, h53AON1 and h55AON, where low levels of exon skipping were detected without or with doses ≤25nM of AONs and an improvement in skipping levels was seen for 50-200 nM (data not shown).

Thereafter, the effect on DMD patient cells with a deletion of exon 51-55 was investigated. For this deletion, the reading frame can be restored by exon 50 skipping. As has been reported previously, there is some very low level of exon 50 skipping detectable in untreated cells^18^. Treatment with increasing concentrations of h50AON1 (fig. 1c) or h50AON2 (data not shown) resulted in a dose-dependent increase of exon 50 skipping. Exon skipping levels were further increased by cotreatment with 6TG. However, 6TG treatment by itself also resulted in similar levels of exon skipping (fig. 1c, most right black bar).

Because exon skipping was observed after 6TG addition in the absence of AONs, other regions of the dystrophin transcript were investigated to assess exon skipping events. The exon 70-75 region is known to be subjected to alternative splicing events in a tissue and differentiation-specific manner. In untreated human control cells exon 71 and 71 to 74 skipping was observed (fig. 1d/e, left lane/bars). However, after 6TG treatment the alternative exon skipping events were greatly enhanced and exon 71, 74, a combination of 71 and 74, and 71-74 skipping were observed (confirmed by sequence analysis (fig. 1d/e)). Furthermore, exon 45, 46, 53 and 55 skipping was observed in the exon 44-55 region (data not shown), suggesting that splicing is disrupted throughout the transcript rather than that 6TG specifically enhances AON-mediated exon skipping.

**Effect of 6TG on muscle cells in vitro.**
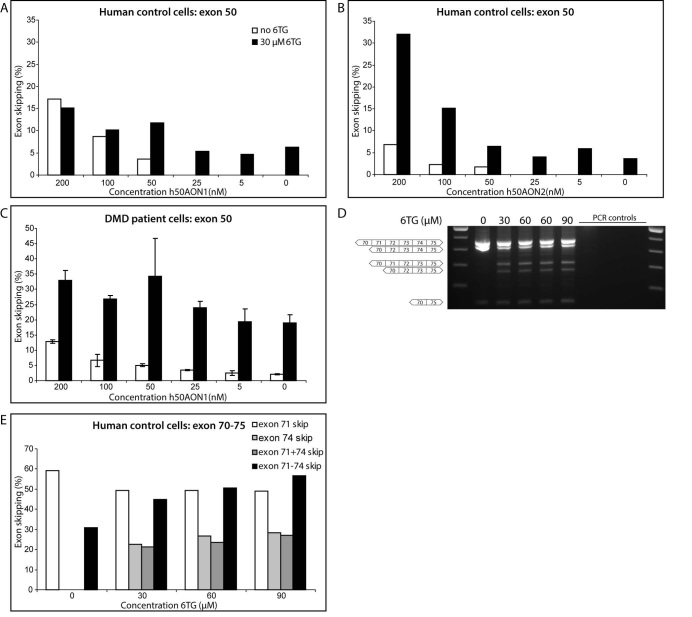
A/B) In healthy control muscle cells, 6TG only enhanced exon skipping with 2OMePS AON at lower concentrations of AON (A) or with a less efficient AON (B). C) In DMD patient cells harbouring a deletion 51-55, skipping of exon 50 was observed after treatment with AONs against this exon. This was greatly enhanced by 6TG treatment. 6TG alone also induced high skipping of this exon (right bar). D/E) Example of greatly enhanced alternative exon skipping in control cells by 6TG around exon 70-75. In non-treated cells next to the wild type product (468 bp) only skipping of exon 71 (429 bp) and 71- 74 (138 bp) was seen, whereas in 6TG-treated cells also skipping of 74 (309 bp) and combined skipping of exon 71+74 (270 bp) was observed.

**6TG enhances mouse exon 23 skipping in cultured mouse cells *in vitro*, but not in mdx mice *in vivo***

The effect of 6TG was then tested *in vivo *by intramuscular injections of 2OMePS mouse exon 23AONs in the gastrocnemius muscles of *mdx *mice on two consecutive days. Mice were sacrificed ten days after the last injection and the injected muscles were isolated and analyzed. Exon 23 skipping was only observed after treatment with 23AON and no enhancement was seen after co treatment with 6TG in any tested concentration (fig. 2a).

Since Hu et al. reported increased exon 23 skipping levels after combinational treatment with a PMO against exon 23 and 6TG compared to PMO alone^15^, the experiment was repeated with murine exon 23AONs of the 2OMePS and the PMO backbone. First the effect of 6TG was explored *in vitro *in mouse C2C12 cells for both chemistries. Here, only a moderate increase of exon 23 skipping was observed after treatment with low concentrations of either AON. The increase was less obvious at higher AON concentrations (fig. 2b). Note that exon skipping levels induced by 2OMePS and PMO AON cannot directly be compared, since for 2OMePS AONs PEI is needed as a transfection reagent (leading to very efficient transfection), while PMOs were added to the medium. Hu et al. did not observe exon skipping after treatment with 60 or 90 μM 6TG by itself. However, our results showed some exon skipping with 60 μM 6TG in the absence of AON treatment (fig. 2b, left white bar).

Thereafter the *in vivo *experiment with local treatment was repeated now using both PMO and 2OMePS AONs. As has been observed previously, PMOs induced more efficient exon 23 skipping than 2OMePS AONs^21^. Again, no increase of exon skipping levels by 6TG was seen for 2OMePS AONs (fig. 2c). Furthermore, in contrast to the results obtained by Hu et al., exon skipping levels by PMO AONs were also not increased (fig. 2c). No induction of exon 23 skipping was observed after treatment with 6TG alone. Finally, the effect of 6TG on dystrophin protein expression was investigated (fig. 2d/e). Untreated *mdx *mice or mice treated with 6TG alone did not have any dystrophin expression. Treatment with PMO and 2OMePS AONs induced dystrophin expression, but combinational treatment with 6TG did not increase the dystrophin levels. In accordance with the higher exon skipping levels, dystrophin levels were higher after PMO than 2OMePS treatment. However, the differences between individual mice were larger as well after PMO treatment.

In summary, 6TG by itself induced numerous exon skipping events *in vitro *at low concentrations and only showed enhancement of exon skipping levels at low AON concentrations and/or of suboptimal AONs. *In vivo *no effect of 6TG on exon skipping levels was observed.

**Effect of 6TG on exon 23 skipping and dystrophin protein expression.**
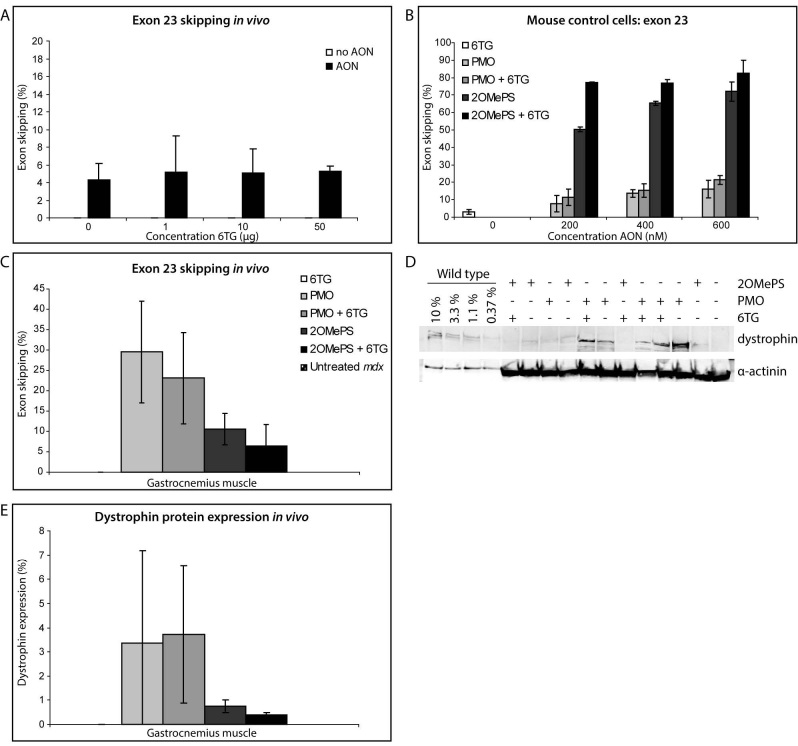
A) *In vivo* in *mdx* mice no enhancement by 6TG of exon skipping by 2OMePS AON targeting exon 23 was observed. No skipping of exon 23 was observed after injection with 6TG alone. B) *In vitro* in a mouse control cell line, 6TG enhanced exon skipping by both 2OMePS and PMO AONs only moderately at low AON concentrations. Without AON treatment no exon 23 skipping was observed. However 6TG alone induced a small percentage of exon 23 skipping by itself (left white bar). C) After local injection *in vivo*, no enhancement of exon skipping by 2OMePS or PMO AON against exon 23 was observed. No skipping of exon 23 was observed after injection with 6TG alone. D/E) No effect of 6TG on dystrophin protein expression was observed by itself or after combinational treatment with AON.

## Discussion

At present only palliative therapies are available for DMD. Exon skipping induced by AONs is the most promising therapeutic strategy that targets the underlying genetic effect and is currently tested in phase III clinical trials^5^. Since higher exon skipping levels will probably lead to enhanced therapeutic effects, several ways to improve efficiency are under investigation. One of the strategies is to combine AON treatment with chemical compounds. 6TG was identified as a compound that could enhance PMO mouse exon 23 skipping *in vitro *and locally *in vivo* 15. To further explore whether the effects of 6TG on exon skipping also apply to other exons and AON chemistries, here additional experiments were performed.

*In vitro *results showed that 6TG indeed had the potential to enhance exon skipping for 2OMePS AONs as well, but only when combined with suboptimal AONs or at suboptimal concentrations. Furthermore, various additional exon skipping events were observed after treatment with 6TG alone in different regions of the dystrophin transcript. By contrast, *in vivo *after local, intramuscular injections no enhancement of AON-induced exon skipping levels or exon skipping events by 6TG alone were observed. This in contrast to previous published results where increased exon skipping was observed after intramuscular PMO and 6TG injections and no exon skipping events were detected after 6TG treatment alone^15^. Our first explanation for this discrepancy was that the enhancing effect of 6TG might be dependent on the backbone chemistry of the AON, which influences the biophysical, biochemical and biological properties (as reviewed elsewhere 22 23 ), but this was ruled out by repeating the experiment with AONs of both chemistries. We could still not reproduce the reported effects for PMOs, despite the fact that the conditions for the experiments were very similar: identical PMOs were used ,the same route of administration was used, mdx mice were of comparable age (6-8 wks old) and time of analysis after injection (2 weeks/ 10 days) was almost identical. The only difference was the specific muscle that was injected (tibialis anterior versus gastrocnemius), but we feel it is unlikely this causes the observed discrepancy, since a correction factor (2.5 times) was used to account for the difference in size of the muscle. Absolute exon skipping percentages cannot be compared between both studies, since no values are reported in the published study and skipping percentages are known to vary with different methods of quantification^20^.

The exon skipping events we observed after 6TG treatment throughout the dystrophin transcript, suggest splicing may have been disrupted for other genes as well. As such, unintended effects of 6TG treatment cannot be ruled out. Furthermore, 6TG is known for its antileukemic effects due to cytotoxicity via several mechanisms after being incorporated in the DNA during replication. This causes miscoding, leading to recognition by the post-replicative mismatch repair system, which cuts at the miscoded base pair^24^ ^25^. Furthermore, the 6TG modified-DNA duplex modifies the structure very locally, which alters specific DNA-processing enzyme activities and recognition by proteins^26^ ^27^. In addition, the presence of 6TG blocks the formation of quadruplex DNA by telomeres and other DNAs, which causes blockage of replication and thereby cytotoxicity after one round of replication^28^. All these effects explain the delayed onset of cytotoxicity by 6TG. These mutagenic effects mainly affects rapidly proliferating cells like leukemic cells^29^, but will also affect other dividing cells. Since 6TG in leukaemia is always used in treatment regimens combining several chemotherapeutic agents, it is difficult to interpret the contribution of 6TG itself to the toxicity observed after chemotherapy in most studies^30^ ^31^. Long-term treatment with 6TG alone in humans or animals has not been reported. However, there are reports about 6-mercaptopurine (6MP, a guanine analogue related to 6TG). Although there is no evidence for secondary malignancies after chronic maintenance chemotherapy with 6MP^32^, repeated treatment of mice with a low dose of 6MP resulted in lethal mutations^33^. Therefore, it is not unlikely 6TG by itself could have similar side effects. This combined with our own observations of disturbed splicing, makes us doubt whether 6TG would be a good candidate to improve the therapeutic efficiency of AON therapy.

However there are other candidates to improve exon skipping levels with more specific effects, like the aforementioned TG003 for exon 31 skipping^13^. Hopefully further research will identify a compound that can improve AON-mediated exon skipping more specifically.

## Correspondence

Corresponding author: Annemieke Aartsma-Rus (A.M.Aartsma-Rus@lumc.nl)
